# Evaluation of antioxidant properties of lemon verbena (*Lippia citriodora*) essential oil and its capacity in sunflower oil stabilization during storage time

**DOI:** 10.1002/fsn3.637

**Published:** 2018-04-02

**Authors:** Reza Farahmandfar, Maryam Asnaashari, Mehdi Pourshayegan, Sara Maghsoudi, Hannaneh Moniri

**Affiliations:** ^1^ Department of Food Science and Technology Sari Agricultural Sciences & Natural Resources University (SANRU) Sari Iran

**Keywords:** antioxidant activity, lemon verbena, oxidation, sunflower oil

## Abstract

In this study, lemon verbena essential oil as a natural antioxidant was used to increase the stability of sunflower oil, and stabilization effects in terms of storage conditions were compared with synthetic antioxidant (BHT). For this purpose, the antioxidant activity of the essential oil was determined by DPPH assay and β‐carotene bleaching method. Then, lemon verbena essential oil (0, 400, 800, and 1,600 ppm) was added to sunflower oil without synthetic antioxidant and stored at 60°C for 60 days. Results from different parameters (peroxide value, free fatty acid, iodine value, total polar compound, carbonyl value, conjugated dienes, and oxidative stability index) were in agreement with each other, suggesting that lemon verbena essential oil (1,600 ppm) could act better than BHT in inhibition of lipid oxidation in sunflower oil and can be used as predominant alternative of synthetic antioxidants.

## 1 INTRODUCTION

Lipid oxidation is a major cause for the deterioration of fat‐containing food. Furthermore, it initiates other undesirable changes in food affecting its nutritional quality, safety, color, flavor, and texture (Ramadan, 2013; Sayyari & Farahmandfar, 2017). Autoxidation of polyunsaturated lipids involves a free radical chain reaction that is generally initiated by exposure of the lipids to light, heat, radiation, metal ions, or metalloprotein catalysts (Eshghi, Asnaashari, Haddad Khodaparast, & Hosseini, 2014). Therefore, the inhibition of free radical autoxidation by antioxidants is of great practical importance in preserving polyunsaturated lipids from deterioration. Synthetic compounds, such as butylated hydroxyanisole (Iqbal and Bhanger), butylated hydroxytoluene (BHT), propyl gallate (PG), and tert‐butylhydroquinone (TBHQ), are widely used antioxidants due to their low cost, high stability, and effectiveness (Asnaashari, Tajik, & Khodaparast, 2015). However, possible toxicological side effects of synthetic antioxidants on human health were reported. The synthetic antioxidants have been widely used to control lipid oxidative rancidity in foods, which is a major cause of quality deterioration, nutritional losses, off‐flavor development, and discoloration. Besides, prolonging the shelf life of food products, these compounds are able to retard the progress of many oxidative stress‐related chronic diseases in human (Farahmandfar, Safari, Ahmadi Vavsari, & Bakhshandeh, 2016). Therefore, dietary antioxidants also have an important role as nutraceuticals due to their role in protecting the body from free radicals, reactive oxygen species, and reactive nitrogen species, which are derived either from normal metabolic processes or from external sources (Farhoosh, Johnny, Asnaashari, Molaahmadibahraseman, & Sharif, 2016). The lemon verbena (*Lippia citriodora*) grows spontaneously in South America and is cultivated in North Africa (Morocco) and southern Europe. In these areas, the leaves are largely used as herbal tea for their aromatic, digestive, and antispasmodic properties (Casamassima et al., 2013). The lemon verbena is a folk remedy for colds, fever, spasms asthma, flatulence, colic, diarrhea, indigestion, insomnia, and anxiety (Bilia, Giomi, Innocenti, Gallori, & Vincieri, 2008; Choupani, Arabshahi Delouee, & Alami, 2014; Felgines, Fraisse, Besson, Vasson, & Texier, 2014). The essential oil from its leaves has been shown to exhibit antimicrobial activity (Kiafar, Zadeh, & Khommami, 2013; Shafiee, Moghadamnia, Shahandeh, Sadighian, & Khodadadi, 2016). Also, antioxidant activity has also been detected in lemon verbena leaves (Felgines et al., 2014), which it has been associated with the presence of flavonoids and phenolic acids (Naser Aldeen, Mansoor, & AlJoubbeh, 2015). The objective of our research was to investigate the antioxidant activity of lemon verbena essential oil. Moreover, oxidative stability of sunflower oil added different concentrations of the essential oil using primary and secondary oxidation products was also determined.

## 2 MATERIALS AND METHODS

### 2.1 Materials

Lemon verbena was obtained from local market (Sari, Iran). All chemicals and solvents were provided from Sigma‐Aldrich (St. Louis, MO) and Merck (Darmstadt, Germany) companies. DPPH (2,2‐diphenyl‐1‐picrylhydrazyl) and β‐carotene were purchased from Sigma‐Aldrich. BHT used as standard antioxidant provided from TITRAN.

### 2.2 Preparation of lemon verbena essential oil

Essential oils were extracted by hydrodistillation from the powdered lemon verbena by the Clevenger‐type apparatus, and the obtained essential oils are stored in a dark container at 4°C until used.

### 2.3 Analysis of lemon verbena essential oil

A GC‐MS instrument (5973N; Agilent Technologies, Wilmington, DE, USA) equipped with a mass selective detector operating in the electron impact mode (70 eV) was used to study the compositions of the lemon verbena essential oil according to Hashemi, Khaneghah, Tavakolpour, Asnaashari, and Mehr (2015). For each compound on the chromatogram, the percentage of peak area relative to the total peak areas from all compounds was determined and reported as relative amount of that compound.

### 2.4 Determination of radical scavenging activity

To evaluate the antioxidant activity of the lemon verbena essential oil, DPPH (1,1‐diphenyl‐2‐picrylhydrazyl) free radical scavenging method was used. DPPH free radicals scavenging were measured by the method described by Farahmandfar, Asnaashari, and Sayyad (2017). Initially, the samples were reacted with the stable DPPH radical in methanol solution. The solution kept for 20 min in the dark, and then its absorbance was measured at 517 nm using spectrophotometer (GBC, Cintra 20). Inhibition of free radical DPPH was calculated using the following equation:

(1) $$I\left(\%\right)=\frac{(A_{\mathrm{blank}}-A_{\mathrm{sample}})}{A_{\mathrm{blank}}}\times 100$$

where *A*_blank_ is the absorbance of the control reaction, and *A*_sample_ is the absorbance of the test compound.

### 2.5 β‐Carotene bleaching method

Oxidation scavenging activity of lemon verbena essential oil was carried out using β‐carotene bleaching method (Bektas, Serdar, Sokmen, & Sokmen, 2016). For this purpose, 5 mg of β‐carotene was dissolved in 10 ml chloroform solvent (high‐performance liquid chromatography grade). Then, 600 μl removed from the prepared solution and were mixed with 40 mg of linoleic acid and 400 mg of Tween 40. Chloroform need to completely evaporate. For this purpose, the rotary vacuum evaporator was used. In the next step, 100 ml distilled water saturated with oxygen was added and gently stirred. 2.5 ml of the solution was transferred in the test tube. Then, 350 μl of each essential oil with a concentration of 2 g/L was added to the test tube. All of the above were performed for blanks. All samples were put into a water bath with temperature of 50°C for 120 min. Then, the samples’ absorbance was read using a spectrophotometer at 470 nm at zero and 120 min. To determine the antioxidant capacity of essential oils, the following equation was used:

(2) $$\text{Antioxidant capacity}\;\left(\%\right)=\frac{\left({\text{DR}}_{\mathrm{blank}}-{\text{DR}}_{\mathrm{sample}}\right)}{{\text{DR}}_{\mathrm{blank}}}\times 100$$

where DR_blank_ is the degradation rate of blank, and DR_sample_ is the degradation rate of sample.

### 2.6 Oxidative stability index (OSI) analysis

Three gram of sunflower oil was mixed separately with different concentrations of the lemon verbena essential oil (Zero, 200, 400, 800, 1,600, and 3,200 ppm) and BHT as control antioxidant and then exposed to Rancimat (Metrohm model 734, Herisan, Switzerland) at 120°C at an airflow of 15 L/hr. During heating process of sunflower oil containing different concentrations of lemon verbena essential oil and BHT at specific time interval, also OSI was determined at 120°C. Measuring vessels, electrodes, connecting tubes, and glassware were cleaned several times before the experiments.

### 2.7 Peroxide value (PV) analysis

The PV (meqO_2_/kg oil) of sunflower oil samples containing different concentrations of lemon verbena essential oil and BHT was measured spectrophotometrically at 500 nm by UV‐VIS instrument (model 160A Shimadzu, Tokyo, Japan). The oil samples were mixed in with 9.8 ml chloroform–methanol (7:3 v/v) on a vortex mixer for 2–4 s. Ammonium thiocyanate solution (50 ml, 30% w/v) and 50 ml of iron (II) chloride solution ([0.4 g barium chloride dihydrate dissolved in 50 ml H_2_O] + [0.5 g FeSO_4_·7H_2_O dissolved in 50 ml H_2_O] + 2 ml 10 mol/L HCl, with the precipitate, barium sulfate, filtered off to produce a clear solution]) were added, respectively, and after adding each of them, the sample was mixed on a vortex mixer for 2–4 s. Then, the absorbance of the sample was read, after 5 min incubation at room temperature (Asnaashari, Farhoosh, & Farahmandfar, 2016).

### 2.8 Acid value (AV) analysis

The AV was determined according to the AOCS (1993) Official Method Cd 3d‐63 (Sayyad & Farahmandfar, 2017). In this method, for determination of free fatty acid, 15 ± 0.01 g of each sunflower oil sample containing different concentrations of lemon verbena essential oil was placed into a 250‐ml Erlenmeyer flask and dissolved in 70 ml reagent‐grade alcohol containing phenolphthalein indicator, and then each oil solution was subsequently titrated with the potassium hydroxide solution.

### 2.9 Iodine value analysis

0.4 g of sunflower oil samples including lemon verbena essential oil placed in iodine flask and 20 ml chloroform was added and dissolved. Then, 20–25 ml of Hanus solution was added and closed the flask completely by parafilm and stand in dark for 30 min. After this time, 20 ml of saturated potassium iodide (15%) and 100 ml distilled water were added and titrated with sodium thiosulfate (0.1 N) solution until yellow color formed and then added 2–3 drops of starch solution and titrated until the blue color is disappeared.

### 2.10 Total polar compounds (TPC) determination

The TPC was determined according to Saoudi et al. (2016) method. At first, silica gel 60, dried (12 hr) at 160 °C, was added five parts of water to 95 parts of it and was shaken vigorously for about 1 min and stay overnight. Then, the silica gel 60 (1 g) was compressed and filled between two cotton wool balls into a pipette tip. Oil sample (500 mg) and toluene (4 ml) were mixed, and finally, the solution (1 ml) was pipetted on top of the pipette tip and used toluene as the eluent. After the solvent was eliminated, weighing TPC% was calculated by the equation of 100 (w−w_1_)/w, in which w and w_1_ are the sample weight and the weight of nonpolar compounds in milligrams, respectively.

### 2.11 Carbonyl value (CV) determination

For CV analysis of sunflower oil samples including different concentrations of lemon verbena essential oil and BHT, one kilogram of 2‐propanol with 0.5 g of sodium borohydride was refluxed for 1 hr to remove any carbonyl components of solvent. Then, 2,4‐dinitrophenylhydrazine (DNPH) (50 g) was dissolved in 100 ml of the solvent including 3.5 ml of 37% HCL. 0.04–1 gr of the oil sample was made by adding the solvent including triphenylphosphine (0.4 mg/ml) to reach 10 ml. Moreover, the solutions of 50–500 μmol/L of 2,4‐decadienal in 2‐propanol were prepared. Then, standard carbonyl compound solution or oil solution (1 ml) and DNPH solution (1 ml) were mixed in a tube. After that, the tube was heated (20 min, 40°C) and cooled in water bath after adding 2% KOH (8 ml). Finally, the absorbance of upper layer after centrifuging (2,000×*g* for 5 min) was read at 420 nm.

### 2.12 Conjugated dienes analysis

The absorbance of sunflower oil samples solutions in hexane (1:600 v/v) was read at 234 nm against a hexane as blank for conjugated dienes.

### 2.13 Statistical analysis

All determinations were carried out in duplicate, and data were subjected to analysis of variance (ANOVA). ANOVAs were performed according to SAS software. Significant differences between means were determined by Duncan's multiple range tests; *p* values less than .05 were considered statistically significant.

## 3 RESULT AND DISCUSSION

### 3.1 Composition of lemon verbena essential oil

The yield of essential oil extracted from lemon verbena with hydrodistillation method is 20.14 ± 0.27% (v/w). The percentage of 15 compounds in lemon verbena essential oil was determined using GC‐MS which was reported in Table 1. Limonene (18.41%), nerol (16.1%), geranial (13.02%), β‐cital (6.94%), and β‐caryophyllene (4.78%) were the most abundant compounds of lemon verbena essential oil.

**Table 1:** Essential oil composition of lemon verbena identified by GC‐MS

Compounds	Percentage (%) ± *SD*
Limonene	18.41 ± 1.24
Sabinene	3.05 ± 0.32
β‐Cital	6.94 ± 1.07
α‐Terpineol	2.03 ± 0.36
β‐Pinene	1.69 ± 0.21
β‐Caryophyllene	4.78 ± 0.47
Curcumene	1.28 ± 0.78
Spathulenol	2.97 ± 0.14
Caryophyllene oxide	2.54 ± 0.67
Neral	16.1 ± 2.97
Geranial	13.02 ± 1.34
β‐Chamigrene	1.17 ± 0.19
β‐Farnesene	1.33 ± 0.27
4‐Phenyl undecan 4‐ol	3.01 ± 1.06
α‐Cedrol	3.41 ± 1.28
Other compounds	18.27 ± 4.09

### 3.2 Determination of radical scavenging activity (DPPH assay)

**Figure 1:**
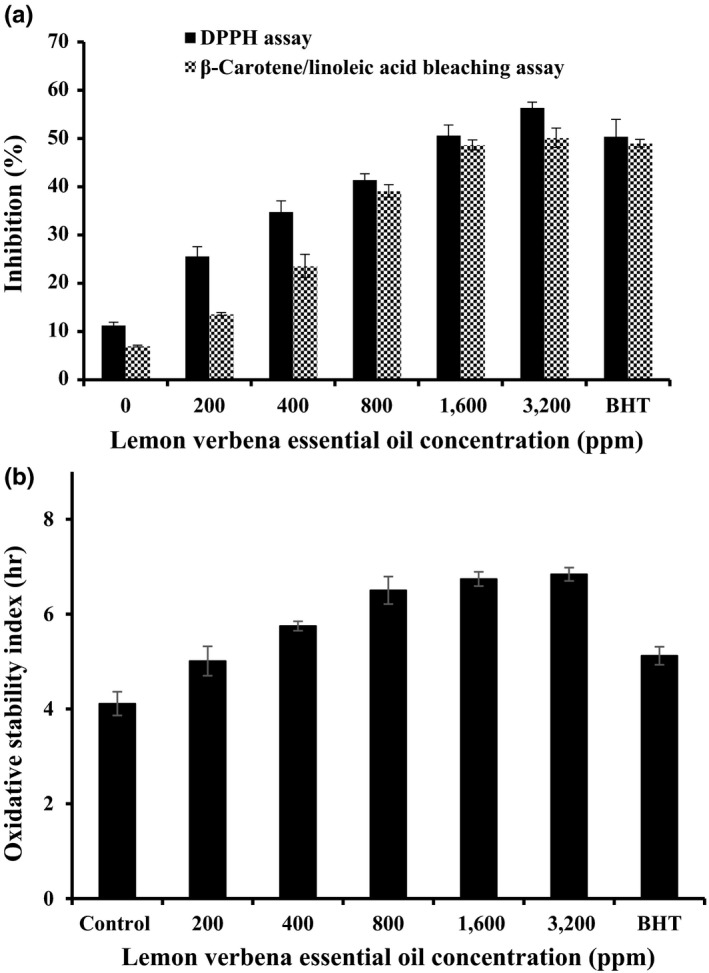
Antioxidant activity of lemon verbena essential oil determination by (a) DPPH assay (black columns) and β‐carotene/linoleic acid bleaching assay (orange columns) and (b) Oxidative stability index (OSI, Rancimat test)

The absorption range of DPPH free radical is maximum within 515–528 nm (Farahmandfar et al., 2017). Immediately after receiving proton from any hydrogen donor, especially from phenolics, it instantly loses its chromophore and becomes yellow (Farahmandfar et al., 2017). As the concentration of antioxidative compounds increases, their DPPH radical scavenging activity also increases and can be defined as antioxidant activity. DPPH radical scavenging test is applied as a rapid method to determine antioxidant activity of chemical substances. Bleaching is known to occur via abstraction of hydrogen by this stable free radical with a maximum absorption band around 515–528 nm and can easily be monitored spectrophotometrically. The antioxidant activity of plant essential oil such as lemon verbena essential oil related to their ability to donate hydrogen atoms or electrons and free electrons (Sharopov, Wink, & Setzer, 2015). DPPH radical scavenging of lemon verbena essential oil was measured in triplicate and is shown in Figure 1. As can be seen, as concentration of the essential oil raised, the DPPH radical inhibition also enhanced. Moreover, the sunflower oil samples including 3,600 ppm of lemon verbena essential oil also act better than BHT in radical scavenging ability. This can be a result of the capability of components within lemon verbena essential oil for scavenging free radicals via electron or hydrogen‐donating mechanisms.

### 3.3 β‐Carotene/linoleic acid bleaching

To indicate the potential antioxidant activity of plant essential oil, synthetic free radical scavenging (DPPH) model will be valuable tool. However, this system does not use a food or biologically relevant oxidizable substrate, so no direct information on an essential oil's protective action can be determined (Kreps, Vrbiková, & Schmidt, 2014). Therefore, assessing the essential oil in β‐carotene/linoleic acid was considered important: water emulsion assay despite its reported limitations. This assay applies that the oxidation of linoleic acid would generate peroxyl free radicals according to the abstraction of hydrogen atom from methylene groups of linoleic acid. The highly unsaturated β‐carotene will be oxidized by the free radicals. The oxidation of β‐carotene will be minimized by the presence of antioxidants in the lemon verbena essential oil. The antioxidants from the essential oil will decompose hydroperoxides formed in this system. So it can be inferred that the degradation rate of β‐carotene is dependent on the antioxidant activity of the essential oils. As can be seen in Figure 1, the oxidation of β‐carotene was reduced due to the presence of antioxidants in lemon verbena essential oil especially in 1,600 and 3,200 ppm. The lemon verbena essential oils possess the potential for scavenging free radicals in a complex heterogeneous medium, according to the β‐carotene/linoleic acid bleaching data in Figure 1.

### 3.4 Oxidative stability index (OSI) of sunflower oil samples including lemon verbena essential oil

Induction period (IP) provides direct evidence for trends in resistance to oxidative rancidity of vegetable oils. The Rancimat analysis aimed to measure the IP by detecting the formed volatile acids during oil oxidation. Test results for oxidative stability index (OSI) are shown in Figure 1. It has been seen that IP of the sunflower oil samples with added lemon verbena essential oil or synthetic antioxidant (BHT) would be significantly larger (*p* < .05) than that of the blank sunflower oils. The samples of lemon verbena essential oil (1,600 and 3,200 ppm) showed an intensive improvement in the OSI value of the sunflower oil in comparison with samples containing synthetic antioxidant (BHT) and control. It was inferred that the lemon verbena essential oil was more effective in stabilizing oil against oxidative deterioration compared to synthetic antioxidants (BHT). Moreover, Table 2 shows the OSI of the sunflower oil samples as affected by different concentrations of lemon verbena essential oil by Rancimat during storage time. All samples of lemon verbena essential oil significantly improved the OSI of the sunflower oil compared to control (1.71 hr) during storage time (60 days). The highest stabilizing effect belonged to 1,600 ppm of the essential oil with 5.94 hr after 60 days, followed by 800 ppm of the essential oil (5.80 hr), 200 ppm BHT (5.67 hr), and 400 ppm of the essential oil (5.37 hr).

**Table 2:** Oxidative stability index (OSI) and iodine value of the sunflower oil as affected by the different concentrations of lemon verbena essential oil (0, 400, 800, and 1,600 ppm) during storage time

Treatments	Storage time (days)				
	0	15	30	45	60
OSI					
Control	4.11 ± 0.14^Ad^	4.14 ± 0.11^Ad^	3.60 ± 0.35^Bd^	2.94 ± 0.23^Cd^	1.71 ± 0.17^Dc^
400 ppm	6.25 ± 0.14^Ab^	6.30 ± 0.14^Ab^	5.99 ± 0.51^Bc^	5.54 ± 0.15^Cc^	5.37 ± 0.08^Db^
800 ppm	6.75 ± 0.17^Aa^	6.80 ± 0.15^Aa^	6.47 ± 0.55^Bb^	5.98 ± 0.16^Bb^	5.80 ± 0.08^Ca^
1,600 ppm	6.74 ± 0.03^Aa^	6.81 ± 0.08^Aa^	6.78 ± 0.28^Ba^	6.25 ± 0.18^Ca^	5.94 ± 0.09 ^Da^
BHT	5.12 ± 0.45^Ac^	5.58 ± 0.58^Ac^	5.37 ± 1.08^Ac^	5.35 ± 0.34^Ac^	5.67 ± 0.78^Aa^
Iodine value					
Control	105.53 ± 2.43^Ad^	105.77 ± 1.48^Ad^	107.43 ± 5.49^Ae^	104.30 ± 2.13^Ad^	104.03 ± 1.13^Ac^
400 ppm	109.20 ± 0.31^Ac^	110.33 ± 1.51^Ac^	112.60 ± 1.81^Ac^	108.88 ± 1.16^Ac^	108.00 ± 1.15^Ac^
800 ppm	109.71 ± 0.32^Ac^	110.84 ± 1.52^Ac^	111.11 ± 1.82^Ad^	109.38 ± 1.14^Ac^	108.50 ± 1.11^Ac^
1,600 ppm	122.70 ± 0.35^Aa^	123.97 ± 1.70^Aa^	124.51 ± 2.05^Aa^	122.33 ± 1.32^Aa^	121.35 ± 1.29^Aa^
BHT	114.67 ± 0.32^Ab^	115.86 ± 1.64^Ab^	114.23 ± 1.91^Ab^	114.33 ± 1.22^Ab^	113.42 ± 1.21^Ab^

Means ± *SD* (standard deviation) within a column with the same lowercase letters are not significantly different at *p* < .05.

Means ± *SD* within a row with the same uppercase letters are not significantly different at *p* < .05.

### 3.5 Peroxide value analysis

**Figure 2:**
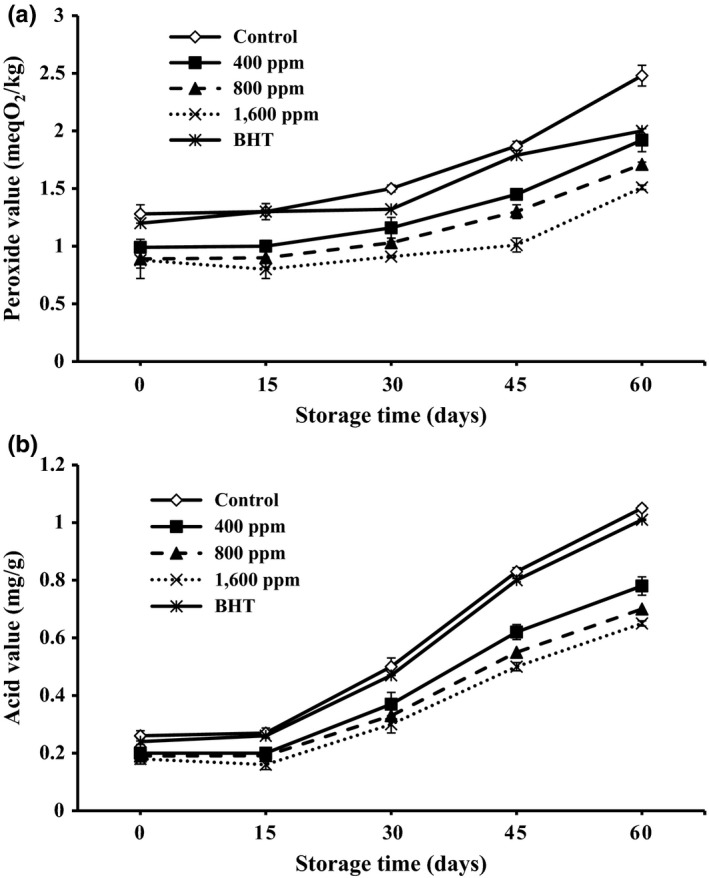
(a) Peroxide value (PV) and (b) Acid value (AV) of the sunflower oil as affected by the different concentrations of lemon verbena essential oil (0, 400, 800, and 1,600 ppm) during storage time. –◊–, Control; □ –■–, 400 ppm of lemon verbena essential oil; ‐▲‐, 800 ppm of lemon verbena essential oil; ··×··, 1,600 ppm of lemon verbena essential oil; –_*_–, BHT (200 ppm)

Peroxides are the main initial products of oil oxidation and can be determined using the peroxide value (PV) (Asnaashari, Hashemi, Mohammad, Mehr, & Asadi Yousefabad, 2015). A higher peroxide value implies a lower oxidative stability (Farahmandfar, Asnaashari, & Sayyad, 2015). The changes in peroxide values are shown in Figure 2. A sharp increase in PV values of all the sunflower oils with and without added antioxidants was observed during the 60‐day storage. The presence of unstable compounds that are susceptible to oxidation was the cause of increase in the PV values. The samples with 800 and 1,600 ppm of lemon verbena essential oil showed a significant decrease (*p* < .05) in PV values than other samples with lemon verbena essential oil (400 ppm) and synthetic antioxidants (BHT) incorporated. As a consequence, oil samples with lemon verbena essential oil incorporated demonstrated significantly lower PV values than that of oils with synthetic antioxidant (BHT) incorporated. Iqbal and Bhanger (2007) investigated sunflower oil stabilization by garlic extract during accelerated storage. Results from different oxidation parameters indicated sunflower oil including 500 and 1,000 ppm of garlic extract has potential to act as a natural antioxidant in sunflower oil stabilization.

### 3.6 Free fatty acids analysis

One important measure of rancidity of foods might be recognized as formation of free fatty acids (Asnaashari, Asnaashari, Ehtiati, & Farahmandfar, 2015). FFA are formed due to hydrolysis of triglycerides and may get promoted by reaction of oil with moisture. The large amount of FFAs, especially low molecular weight ones which have been released by hydrolysis from the glycerides due to moisture, temperature, and lipolytic enzyme reactions, is responsible for undesirable flavors and aromas in oils. The changes in free fatty acids content are shown in Figure 2. During the 60‐day storage, an increase in amount of free fatty acids content of all samples was observed. However, the samples with 400, 800, and 1,600 ppm of lemon verbena essential oil showed a significant (*p* < .05) decrease in FFA values than other samples with lemon verbena essential oil and synthetic antioxidants (BHT) incorporated. So, inhibitory effects of sunflower oil samples with lemon verbena essential oil based on FFA were better than those of BHT. Ramadan (2013) investigated the healthy blends of sunflower oil including (10% and 20%, w/w) of cold‐pressed oils including black cumin oil, cumin oil, coriander oil, and clove oil. Primary and secondary oxidation parameters measurement was made. The results showed the same relationships between PV and AV at termination of storage.

### 3.7 Iodine value analysis

A huge amount of polyunsaturated fatty acids (PUFAs) can be found in sunflower oil, more than 70%. These PUFAs are prone to lipid oxidation. During storage, the double bonds of these PUFAs are attacked by free radicals, which results in the formation of conjugated bonds (Avramović et al., 2015). Hence, measuring the amount of unsaturated fatty acids present in sunflower oil can be used as a reference to determine the freshness of the oil. Adding iodine monochloride to the oil samples can be used for determining the freshness of sunflower oil. The unsaturated fatty acids react with iodine monochloride and release free iodine. The free iodine can then react with sodium thiosulfate (Martínez, Sánchez, Encinar, & González, 2014). The changes in iodine values are shown in Table 2. A decrease in amounts of iodine value of samples including lemon verbena essential oil and samples containing synthetic antioxidant (BHT) was observed between 0 to 60 days. This decrease is indicative of the increase in rate of oxidation due to oxidation of double bonds. Samples including 1,200 ppm of lemon verbena essential oil (121.35) showed the maximum of iodine value during 60 days. The lower IV of sunflower oil including BHT (108.00) compared to lemon verbena essential oils supported the lower efficacy of BHT to protect the oxidation of double bonds of fatty acids in sunflower oil.

### 3.8 Total polar compounds analysis

In here, all of the degradation products without the nonpolar fraction (unaltered triglycerides) will collectively be referred as the total polar compounds (TPC). The changes in total polar compounds are shown in Table 3. These data demonstrated a significant increase in TPCs for all the treatments during storage time. It was concluded from the results that the most stable formulation in terms of the TPCs would be obtained by addition of the lemon verbena essential oil (1,600 ppm). On day 60, higher values of TPCs of the essential oil of lemon verbena are attributed to control with 11.48%, followed by BHT (11.04%), 400 ppm of the lemon verbena essential oil (8.90%). 800 ppm of the essential oil (8.05%), and 1,600 ppm of the essential oil (7.92%).

**Table 3:** Total polar content (TPC) and conjugated diene of the sunflower oil as affected by the different concentrations of lemon verbena essential oil (0, 400, 800, and 1,600 ppm) during storage time

Treatments	Storage time (days)				
	0	15	30	45	60
TPC					
Control	6.70 ± 0.73^Ca^	8.22 ± 0.75^Ba^	10.81 ± 1.16^Aa^	11.18 ± 0.38^Aa^	11.48 ± 0.44^Aa^
400 ppm	5.19 ± 0.56^Bb^	5.23 ± 0.57^Bc^	8.37 ± 0.90^Ac^	8.66 ± 0.29^Ab^	8.90 ± 0.34^Ab^
800 ppm	4.70 ± 0.61^Bb^	4.74 ± 0.62^Bd^	7.56 ± 0.68^Ad^	7.83 ± 0.13^Ac^	8.05 ± 0.47^Ac^
1,600 ppm	4.62 ± 0.50^Bb^	4.66 ± 0.45^Bd^	7.45 ± 0.80^Ad^	7.71 ± 0.23^Ac^	7.92 ± 0.54^Ac^
BHT	6.26 ± 0.65^Ca^	6.75 ± 0.73^Cb^	8.22 ± 0.67^Bb^	10.65 ± 1.33^Aa^	11.04 ± 0.66^Aa^
Conjugated diene					
Control	2.92 ± 0.66^Ca^	4.00 ± 0.61^Bb^	10.69 ± 1.42^Aa^	11.67 ± 2.14^Aa^	11.56 ± 1.51^Aa^
400 ppm	2.69 ± 0.51^Ea^	4.75 ± 0.52 ^Da^	6.71 ± 0.59^Cb^	7.76 ± 0.56^Bc^	8.32 ± 0.18^Ac^
800 ppm	1.87 ± 0.46^Eb^	3.93 ± 0.46^Dc^	5.90 ± 0.53^Cb^	6.94 ± 0.50^Bd^	7.44 ± 0.16^Ad^
1,600 ppm	2.84 ± 0.47^Ea^	3.89 ± 0.21^Dc^	5.86 ± 0.48^Cb^	6.90 ± 0.49^Bd^	7.41 ± 0.12^Ad^
BHT	2.27 ± 0.62 ^Da^	4.01 ± 0.49^Cb^	9.36 ± 1.16^Ba^	10.66 ± 1.08^Ab^	10.57 ± 1.00^Ab^

Means ± *SD* (standard deviation) within a column with the same lowercase letters are not significantly different at *p* < .05.

Means ± *SD* within a row with the same uppercase letters are not significantly different at *p* < .05.

### 3.9 Carbonyl value analysis

**Figure 3:**
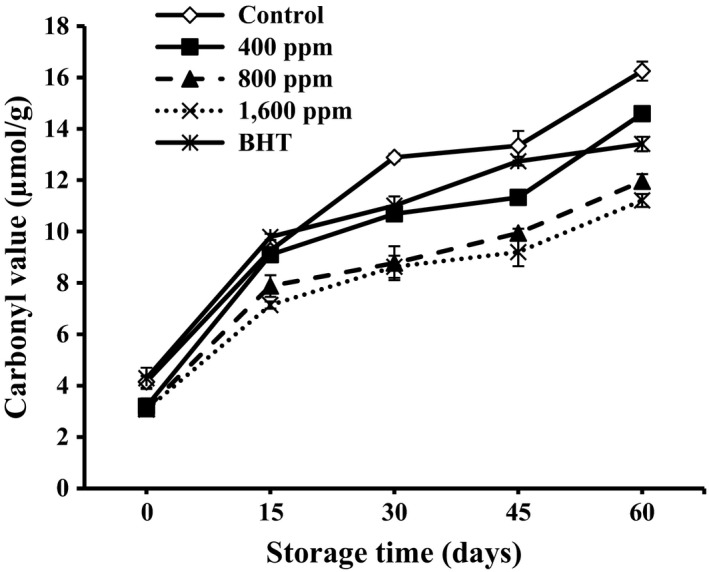
Carbonyl value (CV) of the sunflower oil as affected by the different concentrations of lemon verbena essential oil (0, 400, 800, and 1,600 ppm) during storage time. –♢–, Control; □ –■–, 400 ppm of lemon verbena essential oil; –▲–, 800 ppm of lemon verbena essential oil; ··×··, 1,600 ppm of lemon verbena essential oil; –_*_–, BHT (200 ppm)

Carbonyl compounds in foods can arise from lipid oxidation and particularly during heating (Asnaashari, Farahmandfar, & Kenari, 2016). While lipid oxidation is taking place, primary products transform into secondary products such as carbonyls. Secondary products of oxidation can be estimated by measuring carbonyl value (CV) (Kiralan et al., 2016). The changes in carbonyl values are shown in Figure 3. The oils with 800 and 1,600 ppm of lemon verbena essential oil showed significantly (*p* < .05) lower CV values (11.96 ± 0.53 and 11.21 ± 0.53 μmol/g, respectively) than other samples. It was concluded from the results that 800 and 1,600 ppm of lemon verbena essential oil were more efficient to inhibit oil oxidation than BHT (16.25 ± 0.25 μmol/g). It can be seen that increase in CV in the presence lemon verbena essential oil is slightly lower than sunflower oil samples without any essential oil.

### 3.10 Conjugated diene analysis

Absorbance at 234 nm provides a measure of the content of conjugated dienes (CD), which represent the degree of production of the primary oxidation products (Dridi et al., 2017). The changes in conjugated dienes are shown in Table 3. It is obviously observed that, while the concentrations of lemon verbena essential oil added to the sunflower oil samples are increased, these conjugated diene concentrations decrease. A significant difference was observed in the absorbance of the sunflower oil treated with lemon flower essential oil from the control oil (11.56 ± 1.51) and from the oils with the addition of the synthetic antioxidants (BHT) (10.57 ± 1.00). At the end of storage, oils with 800 and 1,600 ppm of lemon verbena essential oil had minimum value of CD that was 7.44 ± 0.16 and 7.41 ± 0.16, respectively. The effectiveness of lemon verbena essential oil, with the presence of phenolic and tocopherol content, was exhibited in preventing oxidative rancidity along with showing greater stabilizing effect. Hence, there is a higher possibility for replacing synthetic antioxidants. Kiralan et al. (2016) researched on the changes in hexanal, thymoquinone, and tocopherols contents in sunflower including black cumin oils during storage at room temperature. The results indicated that levels of PV, CD, and conjugated triene in sunflower oil and blends increased with an increase in storage time. And stability of blends was better than sunflower oil, due to changes in the amounts of thymoquinone and tocopherols found in black cumin oil.

## 4 CONCLUSION

The presence of bioactive compounds that possess antioxidant properties causes lemon verbena essential oil to inhibit the formation of oxidation products in sunflower oil. Oxidative Stability Index (OSI) test revealed the superiority of essential oil of lemon verbena than the BHT, in preventing of oil oxidation. More reduction in peroxide value, free fatty acid, total polar compounds, carbonyl value, and conjugated dienes in sunflower oil by adding lemon verbena essential oil than BHT was observed by test results. In this study, it is clearly indicated that 1,600 ppm of this essential oil in stabilization of sunflower oil can act as the same effective as BHT. So, lemon verbena may be considered a cheap potential source of bioactive compounds for application as antioxidants in lipid foods.

## CONFLICT OF INTEREST

The authors declare that there is no conflict of interests.
